# DNA Methylation Status of *PAX1* and *ZNF582* in Esophageal Squamous Cell Carcinoma

**DOI:** 10.3390/ijerph14020216

**Published:** 2017-02-22

**Authors:** Jin Huang, Guo Wang, Jie Tang, Wei Zhuang, Li-Ping Wang, Yu-Ligh Liou, Ying-Zi Liu, Hong-Hao Zhou, Yuan-Shan Zhu

**Affiliations:** 1 Department of Clinical Pharmacology, Xiangya Hospital, Central South University, Changsha 410078, Hunan, China; huangjin879288@163.com (J.H.); wangguo32@126.com (G.W.); daratj@163.com (J.T.); yuligh@istat.com.tw (Y.-L.L.); yzliu2005@163.com (Y.-Z.L.); 2 Institute of Clinical Pharmacology, Central South University, Hunan Key Laboratory of Pharmacogenetics, Changsha 410078, Hunan, China; 3 Department of Cardiovascular & Thoracic Surgery, Xiangya Hospital, Central South University, Changsha 410008, Hunan, China; zhuangweihy@sina.com; 4 Department of Clinical Oncology, The First People’s Hospital of Chenzhou, Chenzhou 423000, Hunan, China; wlpwlp2005@126.com; 5 iStat Biomedical Co. Ltd., Taipei 221, Taiwan

**Keywords:** esophageal squamous cell carcinoma, DNA methylation, *PAX1*, *ZNF582*

## Abstract

Hypermethylation of specific gene promoters is an important mechanism of carcinogenesis. A high frequency of promoter methylation of *PAX1* and *ZNF582* genes has been detected in cervical cancer. In the present study, we investigated the methylation status of *PAX1* and *ZNF582* genes in esophageal squamous cell carcinoma (ESCC) tissues. Tumor and paracancerous tissues were obtained from 14 ESCC patients. Genomic DNA was extracted from both tumor and paracancerous tissues, and the concentration of DNA were determined. DNA methylation analysis of *PAX1* and *ZNF582* genes was carried out using quantitative methylation-specific PCR. To assess the diagnostic performance of the two methylated genes for cancer detection, receiver operating characteristic (ROC) curves were generated. Sensitivities and specificities were tested at cut-offs obtained from the ROC curves. The methylation levels of both *PAX1* and *ZNF582* genes were significantly higher in tumor tissues compared to non-tumor paracancerous tissues. The methylation rates of *PAX1* and *ZNF582* in ESCC tumor and paracancerous tissues were 100% and 21.4% (*p* = 0.006), 85.7% and 0% (*p* < 0.001), respectively. The sensitivities and specificities of *PAX1* and *ZNF582* methylation for the detection of cancer were 100% and 85.7%, and 78.6% and 100%, respectively. The DNA methylation levels and frequencies of *PAX1* and *ZNF582* genes were markedly higher in ESCC tumor tissues compared to those in paracancerous tissues. Moreover, the conclusions were verified by using The Cancer Genome Atlas (TCGA) datasets. DNA methylation status of these two genes showed a relatively good sensitivity and specificity for the detection of ESCC tumors. This data suggests that DNA methylation testing holds a great promise for ESCC screening and warrants further prospective population-based studies.

## 1. Introduction

Esophageal cancer (EC) is one of the most aggressive cancers. The incidence and mortality of esophageal cancer are ranked respectively at the ninth and eighth of malignant tumors worldwide [1]. There are two main histological EC types: squamous cell carcinoma (SCC) and adenocarcinoma (AC). These two types have distinct pathogenesis and clinical outcomes. Regional variation in both the incidence and prevalence of common EC types is observed in the world. EC has a high incidence and mortality in China, where it accounts for 70% of the EC cases worldwide, and ranks at the fourth most common cause of cancer related death. More than 90% of patients with EC in China are esophageal squamous cell carcinoma (ESCC). Although significant advances have been made in the diagnosis and therapy of ESCC in the last decades, the overall five-year survival rate for advanced EC is only around 10% due to difficulty in early detection at asymptomatic stage, limited therapeutic weapons and poor prognosis. On the other hand, the five-year survival rate may reach up to 90% if the disease is diagnosed early in the asymptomatic stage. Thus, the understanding of molecular alterations in EC development and the identification of molecular biomarker(s) for EC early detection are crucial in improving EC clinical outcomes and patients’ survival.

As a primary form of epigenetic inheritance, DNA methylation has been extensively studied and widely used for tumor classification, early detection, therapeutic target, and predictive biomarker of metastasis and recurrence. The hypermethylation of CpG islands in the promoter region of tumor suppressor genes, a key mechanism in tumorigenesis, could impede gene transcription and result in a decrease or loss of gene function, a key mechanism in tumorigenesis [2,3,4]. Similar to other cancers, epigenetic silencing of tumor suppressor genes by promoter hypermethylation is a common molecular alteration in ESCC [5,6,7,8,9,10,11,12]. Methylation of CpG islands in the tumor suppressor genes prevents the binding of transcription factors to the corresponding DNA response elements, resulting in a decrease in gene transcription, and ultimately, a loss of tumor suppressing function, leading to an uncontrolled cell growth and tumor development. It has been shown that aberrant methylation of some tumor suppressor genes such as *PTEN*, *SFRP1, RASSF1A*, *DAPK*, *RUNX3*, *UCHL1*, *CDH1*, *p16INK*, *FHIT*, *APC* and *MGMT* [13,14,15,16,17,18] occurs frequently in esophageal cancer. It is therefore the case that alterations in DNA methylation of specific genes may be a useful biomarker for early esophageal cancer detection. However, whether the promoter methylation status of the *PAX1* and *ZNF582* genes is associated with ESCC and could be novel biomarkers for early esophageal cancer detection remains to be elucidated.

Paired boxed gene 1 (*PAX1*) and zinc finger protein 582 (*ZNF582*) are two tumor suppressor genes. Previous studies have demonstrated that the methylation status of *PAX1/ZNF582* may serve as useful biomarkers for the detection of cervical cancer [19,20]. Huang et al. have reported that DNA methylation of *PAX1* gene is a prognostic indicator for oral squamous cell carcinoma [21]. ESCC is homologous to cervical and oral squamous cell carcinoma, in which they belong to squamous cell carcinoma and share similar pathological process. Based on these facts, we hypothesized that alterations in DNA methylation of *PAX1* and *ZNF582* genes were associated with ESCC development and progression, which may serve as a potential biomarker for early ESCC detection.

Using quantitative methylation-specific PCR (qMSP), we demonstrated for the first time that *PAX1* and *ZNF582* genes were aberrantly methylated in ESCC tumor tissues compared to the paracancerous normal tissues, and the levels of DNA methylation were significantly associated with tumor progression. This data suggests that the methylation status of *PAX1* and *ZNF582* genes may be a potential biomarker for ESCC detection.

## 2. Materials and Methods

### 2.1. Patients and Samples

This study was approved by the Institutional Review Board of Department of Clinical Pharmacology, Xiangya Hospital, Central South University (registration number: CTXY-150005-2; date of approval: 2015-11-18) and by Chinese Clinical Trial Registry (registration number: ChiCTR-ROB-15007486; date of approval: 2015-11-29). Tumor and paracancerous tissues were obtained from 14 patients with ESCC, who had surgery to resect the tumor between May and November 2015. Inclusion criteria were as follows: (a) patients with ESCC who needed surgical resection; and (b) cases with a sufficient amount of residual tumor and paracancerous tissues for DNA methylation analysis. All subjects were provided a written informed consent prior to the study. Clinical and pathological features of 14 patients were listed in Table 1. Histopathological examination was performed to characterize the tumor tissues, and three to five tumor samples were taken from each subject. The tumor tissues were defined as the sections next to the tumor diagnosed histopathologically. Paracancerous tissues were taken from surgically dissected tissues approximately 2 cm away from the defined tumor area without tumor invasion by histopathology. The dissected tumor and paracancerous tissues were further evaluated by histological examination. Also, tumor samples were microdissected to eliminate surrounding non-tumoral tissues or immune infiltrates. Tumor samples were considered pure and acceptable for inclusion in this study.

### 2.2. Genomic DNA Preparation

All tissue samples were fixed in phosphate-buffered saline formalin solution and paraffin-embedded for histological examination. Approximately 300 mg of paraffin-embedded tissues were dissolved in 1 mL turpentine and vortexed thoroughly. The sample was then centrifuged at 16,000× *g* at a bench-top centrifuge for 3 min and washed with 70% ethanol 3 times.

Genomic DNA was extracted using iStat Nucleic Acid Extraction kit (iStat Biomedical Co., Ltd., New Taipei City, Taiwan) according to the manufacturer’s standard protocol. DNA concentration was determined using a BioSpec-Nano spectrophotometer. Samples with a DNA yield of more than 500 ng were used for subsequent testing.

### 2.3. DNA Methylation Determination

Bisulfite conversion of DNA samples was carried out using the iStat Bisulfite Conversion Kit (iStat Biomedical Co., Ltd., New Taipei City, Taiwan) following the manufacturer’s manual. Bisulfited genomic DNA was analyzed for methylation status by BioSpec-Nano spectrophotometer following the standard instructions.

*PAX1* DNA Detection Kit and *ZNF582* DNA Detection Kit (iStat Biomedical Co., Ltd., New Taipei City, Taiwan)—simplified technologies which were based on TaqMan technologies for qMSP [22,23]—were used for DNA methylation analysis of *PAX1* and *ZNF582* genes, respectively. PCR analysis was performed in the Lightcycler LC480 real-time PCR system (Roche Applied Science, Penzberg, Germany). The *COL2A* gene was used as an internal reference and analyzed parallelly with each specimen. The crossing point (Cp) value for *COL2A* ≤ 35 was set as the validity indicator for the testing according to the manufacturer’s protocol.

**Figure 1:**
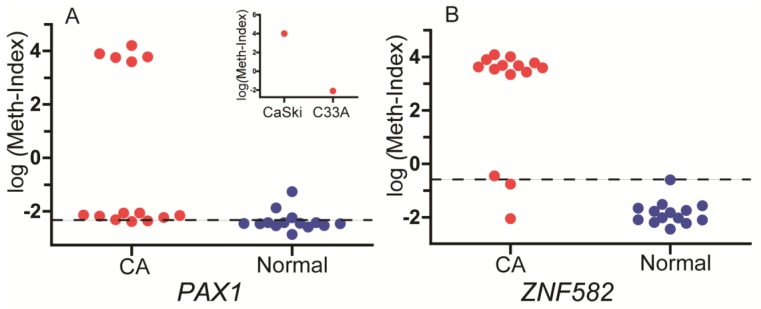
The levels of *PAX1* (**A**) and *ZNF582* (**B**) methylation in cancer (CA) and paracancerous (normal) tissues from 14 esophageal squamous cell carcinoma (ESCC) patients. The level of DNA methylation is presented as log (meth-index). Insets image shows the log (meth-index) of *PAX1* for CaSki and C33A cell lines, which were used as methylation and non-methylation controls, respectively. The cut-off value is shown as a dashed line.

qMSP was done in a 20 μL reaction containing 2 μL of bisulfite converted template gDNA (50 ng), 1 μL of 2× custom detection mix, and 10 μL of 2× custom universal PCR master mix. The reactions were subjected to a pre-incubation at 95 °C for 10 min, followed by 50 cycles at 95 °C for 10 s, and annealing and extension at 60 °C for 40 s. Fluorescence data were collected during the annealing/extension step for the determination of Cp using LC480. For each sample, the PCR allows to detect simultaneously the methylated strands of *ZNF582* or *PAX1* using a FAM-labeled probe, while a probe labeled with VIC amplifies a CpG free region of the *COL2A* gene as internal control, therefore normalizing for the DNA quantity. The DNA methylation levels were assessed as methylation index (meth-index) calculated as follows: 10,000 × 2^(Cp value of gene − Cp value of *COL2A*)^. DNA sample from CaSki cervical cancer cells was used as a methylation control, while DNA sample from C33A cells was used as a non-methylation control as previously described [24] to ensure the quality of bisulfite conversion and qMSP processing. The methylation index for these cell lines were shown in insets image of Figure 1.

### 2.4. Validation Using TCGA Datasets

The datasets of DNA methylation information of 187 esophageal cancer tissues and 16 normal tissues was obtained from The Cancer Genome Atlas (TCGA) database. DNA methylation information were Level 3 datasets and were obtained through an Infinium HumanMethylation 450 BeadChip.

### 2.5. Statistical Analysis

**Figure 2:**
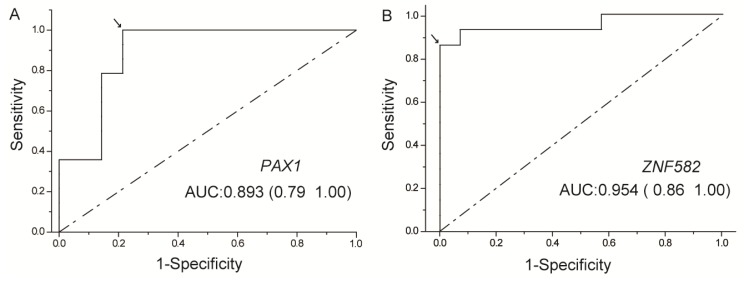
Receiver operating characteristic (ROC) curve analysis of *PAX1* (**A**) and *ZNF582* (**B**). The area under the ROC curve (AUC) of each gene was calculated for the diagnosis of ESCC tumors. The arrow indicates the best sensitivity and specificity.

**Figure 3:**
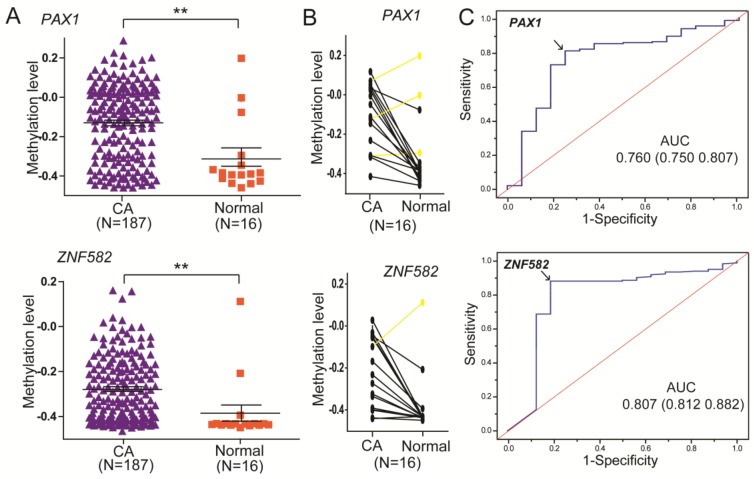
DNA methylation of *PAX1* (cg08156066) and *ZNF582* (cg11740878) analyzed in esophageal cancer samples using The Cancer Genome Atlas (TCGA) dataset. (**A**) The methylation level of *PAX1* and *ZNF582* in 187 esophageal cancer samples and 16 paracancerous samples. ** *p <* 0.05 was considered significant; (**B**) Methylation levels of *PAX1* and *ZNF582* in 16 esophageal cancer samples and their paired normal samples; (**C**) ROC curve analysis of *PAX1* and *ZNF582* for the diagnosis of esophageal cancers from paracancerous tissue.

To evaluate the diagnostic performance of the two methylated genes for cancer detection, receiver operating characteristic (ROC) curves were generated, and the area under the ROC curve (AUC) was calculated for each gene (Figure 2). The cut-off values for *PAX1* and *ZNF582* genes were generated from the 14 subjects with methylation results of paracancerous and tumor tissues from the ROC curves, and the sensitivities and specificities were established using cut-off values determined from the ROC curves. *t*-test was used to compare the methylation levels between the ESCC and paracancerous (normal) samples by using TCGA datasets (Figure 3). The correlation between categorical variables was determined with Fisher exact tests (Table 2 and Table 3). Mann-Whitney U test was used to analyze the correlation of clinical characteristics with methylation level (Table 4). *p* < 0.05 was considered statistically significant. All statistical analysis was performed using SPSS Statistics 19.0 software (IBM Corporation, Armonk, NY, USA). Raw and processed data are stored by the corresponding author of this paper and are available upon request.

## 3. Results

### 3.1. The Levels and Frequencies of DNA Methylation of PAX1 and ZNF582 Genes in ESCC Tumor and Paracancerous Tissues

A total of 14 subjects with ESCC were recruited from Xiangya Hospital located in Changsha, China between May and November 2015. The demographic characteristics of these subjects are presented in Table 1 and Table 3. There were 11 males and three females with a mean age of 57 (age range 45–74 years old), four of them younger than 50 and 10 of them older than 50. DNA methylation of *PAX1* and *ZNF582* genes in both tumor and paracancerous tissues was detected successfully using the current analysis. As shown in Figure 1, the methylation levels of both genes were higher in tumor tissues compared to those in paracancerous tissues. The methylation status of samples and frequencies were determined based on the best meth-index cut-off values calculated using the ROC curves (Figure 2). The cut-off values were shown in Figure 1. Along the lines, log (meth-index) ≥ −2.376 for *PAX1* or log (meth-index) ≥ −0.597 for *ZNF582* were considered to be methylation positivity. The frequency of *PAX1* methylation was 100% (14/14) in the tumor tissues, which is significantly higher than that (21.4%, 3/14) in the paracancerous tissues. The frequency of *ZNF582* methylation in tumor tissues (85.7%, 12/14) is significantly higher than that (0%, 0/14) in paracancerous tissues (Table 2).

Additionally, the results were verified using TCGA datasets. As shown in Figure 3A, the methylation levels of both genes were higher in 187 tumor samples compared to 16 paracancerous samples. The frequency of *PAX1* methylation was 80.7% (151/187) in the tumor samples, which is significantly higher than that (25%, 4/16) in the paracancerous samples. The frequency of *ZNF582* methylation was 88.2% (165/187) in the tumor samples, which is significantly higher than that (18.8%, 3/16) in the paracancerous samples. Besides, as shown in Figure 3B, the methylation level of *PAX1* was higher in tumor samples compared to their paired paracancerous samples in 81.3% (13/16) ESCC patients. The methylation level of *ZNF582* was higher in the tumor samples compared to their paired paracancerous samples in 93.8% (15/16) ESCC patients.

### 3.2. The Sensitivity and Specificity of DNA Methylation Testing of PAX1 and ZNF582 Genes in Distinguishing ESCC Tumors from Non-Tumor Tissues

To evaluate the clinical application, ROC curves were generated and the AUC was calculated for both *PAX1* and *ZNF582* methylation to discriminate tumor from non-tumor tissues (Figure 2). The accuracies of *PAX1* and *ZNF582* methylation testing were 0.893 and 0.954, respectively (Table 2). As shown in Figure 2 and Table 2, the sensitivity and specificity of *PAX1* methylation testing achieved 100% and 78.6% at the best meth-index cut-off value of −2.376, respectively. At the best meth-index cut-off value of −0.597, the sensitivity and specificity of *ZNF582* methylation testing were 85.7% and 100%, respectively.

Additionally, as shown in Figure 3C, the results of the accuracies of *PAX1* and *ZNF582* methylation testing using TCGA datasets were 0.760 and 0.806, respectively (Table 2). The sensitivity and specificity of *PAX1* methylation testing achieved 80.7% and 75.0%, respectively, and the sensitivity and specificity of *ZNF582* methylation testing were 88.2% and 81.2%, respectively.

### 3.3. Association of PAX1 and ZNF582 Methylation to Clinical and Pathological Features of the Patients

The association of *PAX1* and *ZNF582* methylation levels to clinical and pathological characteristics was shown in Table 4. Mann-Whitney U test was used to analyze the levels of gene methylation in the different groups. Unfortunately, most *p* values were above 0.05 due to small sample sizes. However, there is a tendency that *PAX1* methylation level was associated with TNM stage and lymph node metastasis with a *p* value of 0.068 and 0.073, respectively, while *ZNF582* methylation level was associated with carcino-embryonic antigen (CEA) concentration with a *p* value of 0.076 (Table 4). However, there is no association between methylation occurrence frequency of *PAX1* and *ZNF582* and any clinical features as shown in Table 3.

## 4. Discussion

DNA methylation plays a crucial role in the regulation of gene expression and is a potential biomarker for cancer detection. Previous studies have shown that the methylation status of *PAX1* and *ZNF582* genes is a useful testing to distinguish tumor and non-tumor tissues in cervical and oral squamous cell carcinoma [19,20]. In the present study, we have for the first time demonstrated that the levels and frequencies of *PAX1* and *ZNF582* methylation are markedly higher in the tumor tissues compared to non-tumor tissues from ESCC patients, and methylation testing of these two genes has an excellent accuracy and great sensitivity and specificity to detect ESCC tumors, suggesting that *PAX1* and *ZNF582* methylation testing may be a promising biomarker for the detection of ESCC.

*PAX1* gene is a member in the group 1 of the paired box (*PAX*) family of transcription factors, which has four well-defined groups [25]. Members of the *PAX* family typically contain a paired box domain and a paired-type homeodomain, and play critical roles in pattern formation during embryogenesis. *PAX* members have been reported to be overexpressed in several cancers and function in oncogenesis [26,27,28]. On the other hand, *PAX1* and *PAX4* are silenced by DNA methylation in ovarian and cervical cancers and in melanoma, and may function as tumor suppressors [29,30,31]. As a tumor suppressor gene, *PAX1* may be involved in carcinogenesis and tumor progression to invasive or aggressive cancers. Previous studies have reported that *PAX1* methylation testing is a potential biomarker for the screening of cervical cancer with a sensitivity and specificity greater than 80% in the detection of grade III or higher cervical intraepithelial neoplasia (CIN3+) lesions [32,33]. The efficacy of *PAX1* methylation testing in detecting cervical cancer is significantly improved with a combination of cervical cell cytology or HPV 16, 18 genotyping [34,35]. Consistent with the reports in cervical cancers, we observed in the current study that *PAX1* methylation testing had a 100% sensitivity and a 78.6% specificity in the detection of ESCC tumors, indicating that *PAX1* methylation is a valuable biomarker for ESCC diagnosis.

*ZNF582* (zinc finger protein 582) gene, located at chromosome 19q13.43, encodes a protein, ZNF582, in the KRAB-ZNF family, which contains a KRAB-AB domain and nine zinc-finger motifs [22,36]. Most KRAB-ZNF proteins contain the KRAB-AB domain and bind KRAB-associated protein 1 (KAP1) to co-repress target gene transcription. The KRAB-ZNF proteins including *ZNF582* are involved in DNA damage response, proliferation, cell cycle control, and neoplastic transformation [22]. Consistent with the concept that *ZNF582* is a tumor suppressor, *ZNF582* hypermethylation has been reported in acute myeloid leukemia and various invasive cancers [37]. Liou et al. reported that *ZNF582* methylation testing had a 70% sensitivity and an 82% specificity for the detection of cervical cancer CIN3+ lesions [37], and a great sensitivity and specificity in the classification of low-grade squamous intraepithelial lesion (LSIL) [20]. In the present study, we demonstrated that *ZNF582* gene was much more frequently methylated in ESCC tumor tissues compared to non-tumor paracancerous tissues. *ZNF582* methylation testing reaches a 100% specificity and an 85.7% sensitivity for the detection of ESCC tumors (see Table 2), suggesting that it is a useful biomarker for screening ESCC. Moreover, a combination of *PAX1* and *ZNF582* methylation testing could reach a 100% sensitivity and specificity in the detection of ESCC tumors.

To further evaluate the clinical significance of *PAX1* and *ZNF582* methylation in ESCC development and progression, the association of *PAX1/ZNF582* methylation to the clinicopathological features of ESCC patients was analyzed, as a previous report [38] indicated a significant association between *PAX6* methylation and TNM stage of non-small cell lung cancer (NSCLC). In the present study, we did not observe any significant association between *PAX1* or *ZNF582* methylation status and the clinical features analyzed. However, the associations of *PAX1* methylation with TNM stage (*p* = 0.068) and LN metastasis (*p* = 0.073) and *ZNF582* methylation with CEA concentration (*p* = 0.076) appear to be interesting. This observation is in agreement with previous studies in cervical cancer [19,20].

Nowadays, a novel method of swallowing a sponge on a string for esophageal cancer detection has been proposed [39,40,41]. Patients using the cytosponge swallow a small capsule on a string, which expands in their stomach over a few minutes to form a small sponge. The sponge is then gently pulled back out, bringing a sample of cells with it as it comes up through the person’s esophagus. The methylation of *PAX1* and *ZNF582* detected in these cells could act as biomarkers for esophageal cancer screening in the future, which could replace traditional endoscopy as an equally effective but less invasive way of diagnosing for esophageal cancer.

However, it should be noted that there are several limitations in the present study. First, the sample size was small and further studies with a larger sample size are needed to confirm these findings. Second, we could not evaluate the prognostic value of DNA methylation due to the lack of follow-up data. Third, biopsy-matched samples, rather than population-based screening samples, were used to investigate the overall methylation status, precluding the evaluation of its triage potential. Thus, a larger population-based screening or triage trial will determine the actual diagnostic and prognostic performance of DNA methylation in esophageal cancer. Finally, although the two genes used in the current study were selected through extensive literature review, a more comprehensive and unbiased assessment of DNA methylation patterns using genome-wide methylation analysis may provide a better understanding of DNA methylation signatures in ESCC. The elucidation of these DNA methylation signatures will facilitate the development of a more effective strategy for ESCC screening and diagnosis, and accelerate the discovery of potential therapeutic targets for ESCC treatment.

## 5. Conclusions

Our current study demonstrated for the first time that both the levels and frequencies of *PAX1* and *ZNF582* methylation were greatly higher in the ESCC tumor tissues compared to non-tumor paracancerous tissues. *PAX1* and *ZNF582* methylation testing has an excellent accuracy, sensitivity and specificity in distinguishing ESCC tumor tissues from non-tumor tissues. The combination of *PAX1* and *ZNF582* methylation testing could reach a 100% sensitivity and specificity in detecting ESCC tumors, providing a promising biomarker for ESCC screening and diagnosis, although further studies with larger sample size or population-based investigation are necessary to confirm this intriguing finding.

## Figures and Tables

**Table 1:** ESCC tissues of patients with methylation status of *PAX1/ZNF582* genes.

Patient No.	Methylation Status				Age	Gender	TNM Stage	Tumor Size	Tumor HG	Tumor Invasion	LN Metastasis	DM or Recurrence	Family History of ESCC	CEA (mg/mL)
	*PAX1*CA	*PAX1*P	*ZNF582*CA	*ZNF582*P										
1	+	−	+	−	47	Male	IIb	<3 cm	Poor	T2	N1	Negative	Negative	<9.7
2	+	−	+	−	74	Male	IIIc	3–5 cm	Well/moderate	T4	N1	Negative	Negative	<9.7
3	+	+	+	−	58	Male	IIIa	>5 cm	Well/moderate	T2	N2	Negative	Negative	<9.7
4	+	−	+	−	60	Female	IV	>5 cm	Well/moderate	T4	N1	Positive	Negative	>9.7
5	+	−	+	−	52	Male	IIIc	>5 cm	Poor	T4	N2	Negative	Negative	<9.7
6	+	−	+	−	67	Male	IIIa	>5 cm	Poor	T4	N0	Negative	Negative	<9.7
7	+	−	+	−	59	Female	IIa	>5 cm	Well/moderate	T3	N0	Negative	Negative	<9.7
8	+	+	+	−	57	Male	IIIc	3–5 cm	Well/moderate	T2	N3	Negative	Negative	<9.7
9	+	−	+	−	56	Male	IV	3–5 cm	Poor	T4	N2	Positive	Negative	>9.7
10	+	−	−	−	47	Male	IIIc	>5 cm	Poor	T4	N1	Negative	Negative	>9.7
11	+	−	−	−	67	Male	IIIc	3–5 cm	Well/moderate	T4	N1	Negative	Negative	<9.7
12	+	−	+	−	48	Male	IIIa	<3 cm	Well/moderate	T2	N2	Negative	Negative	>9.7
13	+	−	+	−	58	Female	IIIc	3–5 cm	Well/moderate	T2	N0	Negative	Negative	<9.7
14	+	+	+	−	45	Male	IIIc	3–5 cm	Poor	T4	N2	Negative	Negative	<9.7

CA: cancer tissue; P: paracancer tissue; DM: distant metastasis; HG: histological grade; LN: lymph node; CEA: carcino-embryonic antigen.

**Table 2:** Sensitivities, specificities, and AUC of *PAX1* and *ZNF582* methylation in detecting ESCC tumors (*n* = 14).

Detection Modality	Cancer		Paracancer		Sensitivity %	Specificity %	AUC	*p* Value *	95% CI
	U	M	U	M					
*PAX1*	0	14	11	3	100.0	78.6	0.893	<0.001	0.764–1.000
*ZNF582*	2	12	14	0	85.7	100.0	0.954	<0.001	0.871–1.000

* by Fisher exact tests. U: unmethylated; M: methylated; AUC: area under the ROC curve.

**Table 3:** Association of methylation occurrence frequency of *PAX1* and *ZNF582* in cancer tissues to clinical characteristic in 14 ESCC patients (*n* = 14).

Characteristics	Overall Patients (*n* = 14)	Methylated *PAX1*		Methylated *ZNF582*	
		*n*	Percentage	*n*	Percentage
**Age**					
Mean (SD) (range)	57 (8.6) (45–74)				
<50	4	4	100.0%	3	75.0%
>50	10	10	100.0%	9	90.0%
**Gender**					
Male	11	11	100.0%	9	81.8%
Female	3	3	100.0%	3	100.0%
**TNM Stage**					
I/II	2	2	100.0%	2	100.0%
III/IV	12	12	100.0%	10	83.3%
**Tumor size**					
<3 cm	2	2	100.0%	2	100.0%
3–5 cm	6	6	100.0%	5	83.3%
>5 cm	6	6	100.0%	5	83.3%
**Tumor HG**					
W/M	8	8	100.0%	7	87.5%
Poor	6	6	100.0%	5	83.3%
**Tumor invasion**					
T1/2	5	5	100.0%	5	100.0%
T3/4	9	9	100.0%	7	77.8%
**LN metastasis**					
N0	3	3	100.0%	3	100.0%
N1/2/3	11	11	100.0%	9	81.8%
**DM or recurrence**					
Negative	12	12	100.0%	10	83.3%
Positive	2	2	100.0%	2	100.0%
**CEA** (ng/mL)					
>9.7	4	4	100.0%	3	75.0%
<9.7	10	10	100.0%	9	90.0%

DM: distant metastasis; LN: lymph node; HG: histological grade; W/M: well/moderate; CEA: carcino-embryonic antigen.

**Table 4:** Correlation between methylation levels of *PAX1* and *ZNF582* in cancer tissues and clinical characteristics in 14 ESCC patients (*n* = 14).

Characteristics	*PAX1* Methylation Level Log (Meth-Index)		*ZNF582* Methylation Level Log (Meth-Index)	
	Mean ± SD	*p* Value *	Mean ± SD	*p* Value *
**Age**		0.620		0.156
<50	0.763 ± 3.363		1.100 ± 2.788	
>50	−0.370 ± 2.992		2.279 ± 2.360	
**Gender**		0.350		0.755
Male	0.540 ± 3.170		1.851 ± 2.566	
Female	−2.193 ± 0.162		2.276 ± 2.361	
**TNM Stage**		**0.068**		0.715
I/II	−2.304 ± 0.102		3.469 ± 0.177	
III/IV	0.330 ± 3.108		1.688 ± 2.557	
**Tumor size**		0.747		0.651
<5 cm	−0.183 ± 3.172		1.123 ± 2.947	
>5 cm	0.057 ± 3.109		2.556 ± 1.961	
**Tumor HG**		0.897		0.301
W/M	−0.663 ± 2.780		2.131 ± 2.227	
Poor	0.776 ± 3.376		1.690 ± 2.900	
**Tumor invasion**		0.894		0.423
T1/2	−0.938 ± 2.702		2.798 ± 1.823	
T3/4	0.450 ± 3.217		1.466 ± 2.702	
**LN metastasis**		**0.073**		0.876
N0	−2.275 ± 0.123		1.744 ± 3.282	
N1/2/3	0.562 ± 3.149		1.996 ± 2.361	
**DM or recurrence**		0.411		0.522
Negative	0.315 ± 3.122		2.017 ± 2.512	
Positive	−2.210 ± 0.204		1.492 ± 2.746	
**CEA** (ng/mL)		0.887		**0.076**
>9.7	−0.722 ± 2.883		0.123 ± 2.332	
<9.7	0.224 ± 3.174		2.670 ± 2.174	

DM: distant metastasis; LN: lymph node; HG: histological grade; W/M: well/moderate; CEA: carcino-embryonic antigen. * Association analysis for methylation status by Mann-Whitney U test. *p* < 0.1 are indicated in bold.
